# Maternal anemia associated with postpartum hemorrhage: A retrospective cohort in French Guiana

**DOI:** 10.1002/ijgo.70387

**Published:** 2025-07-17

**Authors:** Célia Basurko, Ji‐soo Kim, Alphonse Louis, Milko Sobesky, Allassane Kaba, Mathieu Nacher, Anne‐Sophie Bouthors, Anne‐Christèle Dzierzek

**Affiliations:** ^1^ CIC Inserm 1424 Centre Hospitalier de Cayenne Cayenne French Guiana; ^2^ Amazonian Population Health UA 17 Cayenne French Guiana; ^3^ Department of Anesthesia Centre Hospitalier de Cayenne Cayenne French Guiana; ^4^ Department of Obstetrics and Gynecology Centre Hospitalier de Cayenne Cayenne French Guiana; ^5^ Department of Medical Information Centre Hospitalier de Cayenne Cayenne French Guiana; ^6^ Jeanne de Flandre Academic Woman Hospital, University Hospital Lille France; ^7^ GRITA URL 7365, Lille University Lille France

**Keywords:** antenatal anemia, French Guiana, maternal mortality, postpartum hemorrhage risk factors

Although maternal mortality due to postpartum hemorrhage (PPH) is declining, it remains a global preventable public health priority.^1^ Studies have suggested that maternal anemia may increase the risk of PPH.^2^ However, the criteria for anemia have varied among studies (severe anemia, prenatal or pre‐delivery anemia). In French Guiana, anemia affects 66.7% of pregnant women, and direct causes of maternal mortality still predominate.^3^ Nevertheless, the relationship between anemia and PPH has never been assessed.

A retrospective cohort of women who gave birth at the referral hospital in French Guiana was assembled using a simple random sample from births registered in 2021. The exposure studied was maternal anemia in the first trimester, which was defined as a hemoglobin level less than 11 g/dL. The outcome studied was PPH, which was defined as blood loss of at least 500 mL within 24 hours after delivery. The association between anemia and PPH was examined using a logistic model adjusting for potential confounders such as maternal age, morbid obesity, history of PPH or gestational diabetes, previous vaginal deliveries, multiple gestation, cesarean sections, macrosomia, placenta previa/accreta spectrum or low‐lying placenta, and stillbirth. The hospital's data protection officer approved the research using pseudonymized data from medical records (number 2022‐005; study notices posted in the maternity ward).

Of 3906 deliveries, 422 women were included in the analysis. The age, place of birth, and place of residence of the sample was comparable to the reference population. Most women in the study were aged between 20 and 34 years, foreign‐born, and had less than a high school education. The estimated rate of PPH was 11.6% (95% confidence interval [CI] 8.7–15.0). After controlling for confounders, a significant association was found between first‐trimester anemia and PPH, with an adjusted odds ratio of 2.32 (95% CI 1.11–4.82) (Table 1).

**TABLE 1:** Association between maternal anemia in the first trimester of pregnancy and postpartum hemorrhage (PPH), ANEMOBS study, 2021 (logistic multivariate model)^a^.

Maternal anemia	No PPH (*n* = 373)	PPH (*n* = 49)	Unadjusted (*n* = 422)			Adjusted^b^ (*n* = 400)		
			OR	95% CI	*P* value	OR	95% CI	*P* value
First trimester								
Yes	81 (21.7)	18 (36.7)	2.09	1.11–3.93	0.022	2.32	1.11–4.82	0.024
No	292 (78.3)	31 (63.3)	1 (Ref.)			1 (Ref.)		

Abbreviations: CI, confidence interval; OR, odds ratio.

^a^ Data are presented as number (percentage) unless otherwise stated.

^b^ Adjusted for maternal age ≥ 35 years (*P* = 0.905), morbid obesity (*P* = 0.035), history of PPH (*P* = 0.060), history of gestational diabetes mellitus (*P* = 0.255), more than four previous vaginal deliveries (*P* = 0.475), multiple gestation (*P* = 0.142), cesarean sections (*P* = 0.002), macrosomia (*P* = 0.039), placenta previa/accreta spectrum or low‐lying placenta (*P* = 0.016), and stillbirth (*P* = 0.021).

After adjusting for confounding, our study confirmed the initial hypothesis. The hypothesized pathophysiologic mechanism of the association between anemia and PPH may be related to excessive nitric oxide production by the trophoblast and placenta.^4^ Further studies are, however, needed to understand the underlying pathophysiologic mechanisms. These results provide additional evidence of the importance of detecting and treating anemia early during pregnancy. This is a critical lever in reducing the incidence of PPH and preventing direct maternal mortality. In the context of widespread nutritional deficiencies, food insecurity, and social health inequalities in French Guiana, vigilance and management of anemia during the early stages of pregnancy seem crucial.

## AUTHOR CONTRIBUTIONS

CB, JSK, and ACD designed and conducted the research. JSK, ACD, AL, MS, and AK were involved with data collection. CB, JSK, MN, ACD, and ASB analyzed and interpreted the data. All authors made contributions to the draft and revision of the article.

## CONFLICT OF INTEREST STATEMENT

The authors have no conflicts of interest.

## Data Availability

The data that support the findings of this study are available on request from the corresponding author. The data are not publicly available due to privacy or ethical restrictions.
